# The Spanish Version of the Short Warwick–Edinburgh Mental Well-Being Scale (SWEMWBS): Evidence on Validity, Reliability, and Test of a Processual Model of Physicians’ Well-Being

**DOI:** 10.3390/healthcare13222855

**Published:** 2025-11-10

**Authors:** Maria A. Andreu, Javier Sánchez-Ruiz, Noemí Sansó, Laura Galiana

**Affiliations:** 1 Espai Vida Medical Center, Private Practice, 07500 Manacor, Spain; maandreusunyer@gmail.com; 2 Advanced Research Methods Applied to Quality of Life Promotion (ARMAQoL), Department of Methodology for the Behavioral Sciences, University of Valencia, 46010 Valencia, Spain; jasan4@alumni.uv.es; 3 Balearic Islands Health Research Institute (IDISBA), Department of Nursing and Physiotherapy, University of the Balearic Islands, 07010 Palma, Spain

**Keywords:** well-being, compassion for others, professional quality of life, emotional distress, healthcare professionals

## Abstract

Background/Objectives: The first aim of this manuscript is to examine the psychometric properties of the Spanish version of the Short Warwick–Edinburgh Mental Well-being Scale (SWEMWBS) in a sample of Spanish physicians. The second aim is to analyze how physicians’ levels of compassion for others, professional quality of life, depression, anxiety, and stress are related to well-being. Methods: The study is part of a randomized controlled trial with a mixed design. The inclusion criteria for participation in the study were: (a) physicians registered in Spain; (b) currently working in Spain; and (c) those who voluntarily agreed to participate. A total of 221 medical doctors were enrolled in one of the three experimental conditions. Results: The confirmatory factor analysis showed an adequate fit. Cronbach’s alpha (0.83) and McDonald’s omega (0.89) provided evidence of reliability. Finally, when the mediational model predicting physicians’ well-being was tested, the examination of the modification indices indicated an unmodeled relationship. A second model was tested, resulting in a better-fitting model. Physicians’ levels of compassion for others significantly predicted compassion satisfaction. Professional quality of life also predicted physicians’ emotional states. Finally, well-being was predicted by depression and stress, and a direct effect of compassion was also found. Conclusions: The SWEMWBS shows potential to become a standard measure of well-being in the Spanish language. Regarding the prediction of well-being, it seems clear that interventions based on compassion should have a place in physicians’ education and workplace settings.

## 1. Introduction

Mental well-being is of the utmost importance to overall health and quality of life. In this sense, positive mental health enables individuals to cope with stressors, realize their abilities, and make meaningful contributions to their communities [1,2]. Furthermore, mental health can be considered an integral part of overall well-being, as it underpins our ability to make decisions and build and maintain relationships [2].

Although relatively understudied until recently [3], the field of positive mental health has gained increasing attention during the past decade [4]. This may be partly due to the fact that mental well-being has been linked to important health outcomes such as lower risks of developing mental and physical disorders, reduced disability, and decreased use of health services [5,6,7,8].

### 1.1. Physicians’ Well-Being

In the case of healthcare workers, well-being has been associated with a myriad of variables such as occupational stress and burnout, self-compassion, anxiety, and depression, to name a few [9,10,11,12,13,14]. In this sense, Malik and Annabi [15] suggested that psychological distress and burnout can have a significant effect on the mental well-being of healthcare professionals. Indeed, burnout [16], together with anxiety and depressive symptoms [17,18], has been associated with physicians’ working conditions and heavy workloads. In light of these findings, several authors have proposed that working conditions and organizational stress can lead to compassion fatigue and burnout, making it more likely for depression and anxiety to emerge, which, in turn, are indicators of poor mental well-being [19].

These relationships among the aforementioned variables (i.e., stress and burnout, depression, anxiety, and mental well-being) are particularly important, as recent studies on physicians’ health suggest that their levels of depression, anxiety, and burnout have significantly increased since the COVID-19 outbreak [20,21]. For example, Aiken et al. [22] found that 32% of physicians reported poor work–life balance, 32% showed signs of burnout, and around 4-10% intended to leave their position in the near future. In a systematic review, Jachmann et al. [23] found that depressive symptoms ranged from 15.5% to 19.3%, with burnout rates varying from 18% to 71.4%, among emergency department physicians and nurses. Finally, regarding anxiety, John et al. [14] reported that one-fourth of physicians suffered from it (25.8%). 

The presence of various stressors takes a toll on the mental health of healthcare workers [9], with professional quality of life and compassion being cited as possible buffers against this stress [9,10,12]. Indeed, some studies have found that physicians’ professional quality of life, including burnout, as well as the dimensions of compassion satisfaction and compassion fatigue, can predict their depressive, anxious, and psychosomatic symptomatology [24]. Moreover, healthcare professionals’ professional quality of life levels have been shown to mediate the effect of organizational support (or the lack thereof) on well-being [25].

Among this complex set of variables, an important body of recent research has focused on the role that compassion for others may play. Compassion is generally understood as the awareness of another person’s suffering and the desire to alleviate it [26,27,28,29]. Unlike empathy or empathic distress, compassion is not a self-oriented response to another person’s suffering, accompanied by a desire to withdraw from the situation to avoid negative feelings, but rather a feeling of concern for another person’s suffering, accompanied by a motivation to help [30]. Focusing on insights into compassion, evidence has shown that healthcare professionals’ levels of compassion for others may enhance their ability to receive social support from peers [31], leading to more adaptive strategies for coping with stress, which, in turn, could result in greater well-being, as found by Kiser et al. [32]. Some authors, such as Alcaraz-Córdoba et al. [33], have even suggested that compassion fatigue among healthcare professionals may stem from a deficit in compassionate skills. Moreover, compassion for others seems to play a key role in job engagement and satisfaction when it comes to healthcare personnel [34]. In fact, García-Campayo et al. [35] have recently found that compassion for others is correlated with well-being even after controlling for numerous sociodemographic variables. Nevertheless, other authors have argued that, under excessive environmental demands, high levels of compassion for others can increase the likelihood of suffering greater burnout as individuals under stress tend to expend more resources in helping others, which can lead to exhaustion and diminished well-being [34].

### 1.2. Well-Being Measurement

Traditionally, as cited by VanderWeele et al. [36], the scientific literature has identified at least three main domains into which well-being can be divided, namely hedonic, evaluative, and eudaimonic [37]. The first of these (hedonic) defines the well-being of a person based on the number of positive and/or negative experiences they have [36]. In this sense, individuals who experience a higher proportion of positive emotions will have greater well-being than someone whose predominant affective states are negative [35]. Secondly, evaluative well-being is closely associated with how satisfied a person is with their life [36]. Finally, as noted by VanderWeele et al. [36], eudaimonic well-being focuses on “whether individuals feel they have attained self-realization, or if they are fully functioning or fulfilling a sense of purpose” (p. 2).

Based on this division, there are numerous instruments that aim to measure well-being, with most of which assess different aspects or perspectives of the construct [3,36]. Some of the most notable are: the Satisfaction With Life Scale (SWLS) [38], which evaluates mainly the cognitive/evaluative perspective [3]; the Positive Affect and Negative Affect Scale (PANAS) [39] and the Scale of Positive and Negative Experiences (SPANE) [40], which focus on the hedonic perspective; the Ryff’s scales of Psychological Well-Being (RPWB) [41], which center on around the eudaimonic point of view; and, the WHO Well-being Index (WHO-5) [42,43], which aim to measure both physical and mental health. Nevertheless, despite the recognition and widespread use of these instruments, some criticism has emerged. For example, regarding the use of the PANAS, SPANE, SWLS, and RPWB in the field of mental well-being research, some authors suggested that measuring mental well-being as a whole should not rely solely on these instruments, as constructs such as life satisfaction and hedonic well-being are not equivalent to the general sense of mental well-being [44]. In the same vein, some authors suggest that measures should include both hedonic and eudaimonic-centered items to provide a more comprehensive understanding of the concept of mental well-being [45]. When it comes to the WHO-5, for example, some authors have pointed out that it does not adequately capture negative emotions [46,47].

For these reasons, such instruments tend to offer a limited view of the broad construct of well-being, which may have contributed to the lack of consensus on a “gold standard’’ measure for it [48]. Moreover, several authors, including Su et al. [49] and Lindert et al. [50], have suggested that more comprehensive instruments integrating the eudaimonic, hedonic, and evaluative perspectives might be more valuable for policymakers and healthcare systems, as they assess a broader range of potential factors influencing overall well-being while also being shorter than the combination of several more specific scales. 

With this diversity in mind, Tennant et al. [3] sought to develop an instrument that could improve upon previous scales and capture a broader perspective of well-being, including items related to affect-emotions (hedonic), cognitive-evaluative perceptions, and psychological functioning (closely aligned with eudaimonic point of view), in a short form suitable for use in population surveys [3]. The result of this effort, which combined qualitative and quantitative research, was the Warwick–Edinburgh Mental Well-Being Scale (WEMWBS) and, later, its shortened version, the Short Warwick–Edinburgh Mental Well-being Scale (SWEMWBS) [3,4]. To date, the WEMWBS and its brief counterpart, the SWEMWBS, have received considerable attention, having been used in more than 50 countries (both Western and Non-Western) [51], translated into multiple languages, such as Danish [52], Spanish [48], Arabic [53], and Chinese [54], and applied in both general and patient populations, as well as in national health surveys (e.g., the National Health Service in the United Kingdom). This is partly due to the strong psychometric properties demonstrated by the WEMWBS and the SWEMWBS over the years, including a robust unidimensional factorial structure and reliable scale scores that have been tested across different samples, instrument versions, and study designs [55]. Indeed, qualitative studies have shown that users tend to prefer the SWEMWBS over other instruments for assessing their mental health [56,57]. 

### 1.3. Aims of the Study

As acceptance and use of the SWEMWBS continue to grow due to its strengths, it is important to provide evidence on the use of the SWEMWBS in its Spanish version. The first aim of this manuscript is therefore to examine the psychometric properties of the Spanish version of the Short Warwick–Edinburgh Mental Well-being Scale in a sample of Spanish physicians. Specifically, the WEMWBS has proven to be a useful tool for measuring well-being among physicians in China [58], Pakistan [59], and Great Britain [60]. However, there is currently no evidence regarding the performance of the shortened WEMWBS among physicians, nor in its Spanish version. The second aim is to analyze how physicians’ levels of compassion for others, professional quality of life, depression, anxiety, and stress are related to well-being. Finally, we sought to gain a better understanding of how well-being among healthcare workers may be influenced by these predictors by testing a process-based model of physicians’ well-being, grounded in both well-being theories and previous empirical evidence.

## 2. Materials and Methods

### 2.1. Adaptation Procedure and Study of Content Validity

The scale was not translated into Spanish because a Spanish version of the Warwick–Edinburgh Mental Well-Being Scale (WEMWBS) already existed [45]. Therefore, to create the short version of the questionnaire, the seven items comprising the SWEMWBS, as published by Stewart-Brown et al. [4], were selected from the full-length Spanish WEMWBS presented by López et al. [48]. 

Once the Spanish items of the SWEMWBS were selected, the Spanish version was presented to four well-being experts for evaluation. All experts were women, aged between 30 and 56 years. Their areas of expertise were diverse: one specialized in well-being measurement, another in well-being across the life cycle, one in organizational well-being, and the last in clinical well-being. The document included an introduction to the task, a description and instructions for the questionnaire, the seven items of the scale with the response format, and space for comments. Experts were asked whether each item was “necessary, useful, and essential to measure mental well-being”, assigning a score of 1 for agreement and 0 for disagreement.

Based on their responses, shown in Table 1, the Content Validity Ratio (CVR) [61] for each item was calculated, ranging from 0.50 to 1. According to Lawshe, a level of 50% agreement ensures some degree of content validity; therefore, CVR values were considered adequate. Subsequently, the Content Validity Index (CVI) was calculated for the entire instrument. The CVI, representing the average of the CVR values for the retained items, was 0.86, providing evidence of the content validity of the Spanish version of the SWEMWBS.

### 2.2. Study Design and Setting

The study is part of the research project Compassionate Look (Mirada Compasiva), a randomized controlled trial with a mixed design, including a between-subjects factor (condition, with three levels: Compassion Cultivation Training Workshop [CCTW], Mindfulness-Based Intervention [MBI], and no-intervention group [NI]) and a within-subjects factor (time, with three levels: pre-training, post-training, and follow-up).

The data collection procedure lasted 10 months, from June 2023 to March 2024. The study began in June 2023 with the submission and subsequent approval of the project by the Ethics Committee of the University of the Balearic Islands. Subsequently, information about the study was disseminated through the College of Physicians and various health centers across the Balearic Islands (Spain). A website was created, where individuals interested in participating could register using an online form. In September 2023, participants were randomly assigned to one of the intervention groups. In October 2023, data from the pre-intervention wave were collected. The intervention was conducted during October and November 2023, with the post-intervention assessment taking place immediately after the last session. Three months later, in February 2024, the follow-up assessment was carried out.

In this study, we used data from the pre-intervention time point. Thus, this specific manuscript relies on cross-sectional data collected in October 2023, before any intervention was administered to the participants.

### 2.3. Participants 

The inclusion criteria for participation in the study were as follows: (a) physicians registered in Spain; (b) currently working in Spain; (c) those who voluntarily agreed to participate in the study. The exclusion criteria in the present study were retired physicians. These criteria were applied to all participants, regardless of the group to which they were subsequently randomly assigned.

For sample size determination, the meta-analysis conducted by Salvado et al. [62] was used as a reference. The authors reported a medium effect size for emotional exhaustion and small effect sizes for depersonalization and personal accomplishment, with the smallest expected difference being ±0.34. This mean difference was converted to an F value, yielding an approximate value of 0.17. To detect this effect size, considering the type of analysis to be used, a mixed multivariate analysis of variance (MANOVA) with three dependent variables (emotional exhaustion, depersonalization and personal fulfillment), a within-subjects factor (time, with three temporal measurement points: pre-intervention, post-intervention, and follow-up) and a between-subjects factor (group, with three categories: CCTW, MBI and NI), and assuming an alpha level of 0.05, a power of 0.95, and a correlation of 0.50 between measures, the required sample size was estimated to be *n* = 140. Taking into account that mindfulness-based interventions (MBIs) typically show experimental attrition rates above 20% [63], a 25% dropout rate was estimated between each measurement point. Thus, to ensure that 140 participants completed the intervention and the follow-up assessment, an additional 25% of participants were added at the post-intervention time point (*n* = 187.5), and another 25% at the pre-intervention time point (*n* = 250). Therefore, the calculated sample size at pre-intervention was *n* = 250.

### 2.4. Measures

The study included several variables, with the outcomes used in this paper being as follows:

- Short Warwick–Edinburgh Mental Well-being Scale (SWEMWBS) [4]. This scale was derived from the original Spanish translation proposed by López et al. [48]. To obtain a shorter version, the same items selected by the original authors (as described by Stewart-Brown et al. [4]) were used. Accordingly, seven items were selected from the long-form Spanish validated version to create the Spanish Short-Warwick Edinburgh Mental Well-being Scale (SWEMWBS). The SWEMWBS uses a 5-point Likert scale, with response options ranging from ‘Never’ (1) to ‘Always’ (5). For the total score, the mean score of the seven items needs to be calculated. Reliability estimates for this scale are reported in the Results section.
- Depression, Anxiety and Stress Scale (DASS-21) [64]. This scale was originally developed by Lovibond and Lovibond [64] as a shortened version of the 42-item DASS. The first Spanish translation was carried out by Daza et al. [65]. The DASS-21 is structured as a three-factor measure that assesses symptoms of depression, anxiety, and stress experienced during the past week using a 4-point Likert scale with response options ranging from ‘Did not apply to me at all’ (0) to ‘Applied to me very much or most of the time’ (3). Reliability estimates in this sample were 0.82 for depression, 0.78 for anxiety, and 0.87 for stress.
- Short Professional Quality of Life (Short ProQOL) [66]. Translated into Spanish by Galiana et al. [66], the short ProQOL was designed by selecting items from the original ProQOL versions 4 and 5 developed by Stamm [67,68]. This scale consists of three dimensions (compassion satisfaction, compassion fatigue, and burnout), each measured with three Likert-type items rated from (1) ‘never’ to (5) ‘very commonly’. In this sample, reliability estimates were 0.83 for burnout, 0.82 for compassion fatigue, and 0.84 for compassion satisfaction.
- Sussex-Oxford Compassion for others Scale (SOCS-O) [64]. The SOCS–O assesses five dimensions of compassion for others by using 20 items, with four items per dimension: (a) recognizing suffering, (b) understanding the universality of suffering, (c) feeling compassion for the person who is suffering, (d) tolerating uncomfortable feelings, and (e) motivation to act or acting to alleviate suffering [69]. Items are rated on a 5-point Likert-type scale ranging from (1) ‘not at all true’ to (5) ‘always true’. For this study, the Spanish version of the SOCS-O translated by Sansó et al. [70] was used. In this sample, reliability estimates were 0.87 for recognizing suffering, 0.87 for understanding the universality of suffering, 0.80 for feeling compassion for the person who is suffering, 0.72 for tolerating uncomfortable feelings, and 0.76 for motivation to act or acting to alleviate suffering.

### 2.5. Data Analyses

Data analyses included the description of the sample and analyses addressing the two aims of the manuscript.

#### 2.5.1. Participants’ Description

Participants were described in terms of gender, age, medical specialization, and average professional experience.

#### 2.5.2. Descriptive Statistics of the Spanish Version of the SWEMWBS

To examine the psychometric properties of the Spanish version of the SWEMWBS (aim 1), we first calculated descriptive statistics for both the individual items and the total scale score. These included the mean, standard deviation, minimum and maximum scores, skewness, and kurtosis. All univariate skewness and kurtosis values for the variables analyzed were satisfactorily within conventional criteria for normality (−3 to 3 for skewness and −10 to 10 for kurtosis), according to the guidelines suggested by Kline [71].

#### 2.5.3. Internal Structure of the Spanish Version of the SWEMWBS

To further address aim 1 and to provide evidence of the internal structure of the scale, a confirmatory factor analysis (CFA) was hypothesized, estimated, and tested, in which one general factor of mental well-being explained the seven items of the scale. To assess the model fit, several indices were used: the chi-square statistic, the Comparative Fit Index (CFI), the Standardized Root Mean Square Residual (SRMR), and the Root Mean Square Error of Approximation (RMSEA). The cut-off criteria for good model fit were as follows: a CFI of 0.90 or above (better of 0.95 or above) and SRMR or RMSEA values of 0.08 or below (better of 0.06 or below) [72]. Specific relationships within the model, including factor loadings, were also examined. In addition, and following the recommendations of Kline [65], modification indices were checked to ensure the absence of unmodeled relationships, and a visual inspection of the residuals was performed. The model was estimated using the Weighted Least Squares Mean and Variance-corrected (WLSMV) method, according to the ordinal nature of the data [73,74,75].

#### 2.5.4. Internal Structure of the Spanish Version of the SWEMWBS 

Also addressing aim 1, we examined the reliability of the scale, including internal consistency estimates for the items (homogeneity and alpha if item deleted) and for the total scale (Cronbach’s alpha and McDonald’s omega).

#### 2.5.5. Process-Based Model of Physicians’ Well-Being Using the SWEMWBS

**Figure 1:**
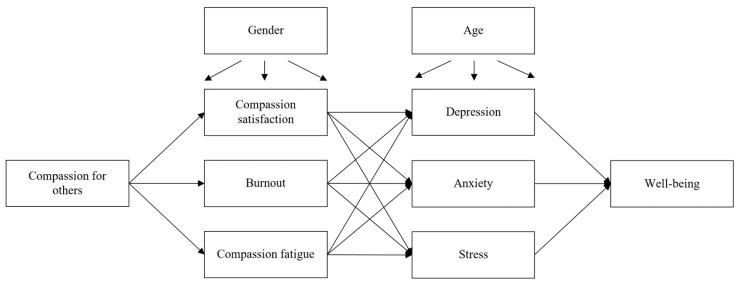
Hypothesized model for the processes affecting physicians’ well-being. Notes: Gender and age were included as control variables.

To address aim 2, studying how physicians’ levels of compassion for others, professional quality of life, depression, anxiety, and stress are related to well-being, we hypothesized a mediational model in which compassion predicted the three dimensions of professional quality of life assessed by the ProQOL, namely compassion satisfaction, burnout, and compassion fatigue. These dimensions, in turn, were expected to predict emotional states of depression, anxiety, and stress as assessed by the DASS-21, which subsequently predicted well-being. Gender and age were included as control variables, predicting all previously mentioned variables. Figure 1 illustrates the hypothesized process-based model of physicians’ well-being. The same fit indices—chi-square statistic, CFI, SRMR, and RMSEA—were used to assess model fit, applying the previously described cut-off criteria, along with the examination of potential unmodeled relationships and a visual inspection of the residuals.

Indirect effects were also calculated, and confidence intervals (CIs) around the estimated effects were obtained using a bootstrap resampling method. This procedure has been recommended as one of the most robust approaches for generating the sampling distributions required for testing indirect effects [76]. The model was estimated using the Maximum Likelihood with robust standard errors (MLR).

All statistical analyses were conducted using SPSS version 26 [77] and Mplus version 8.4 [75].

## 3. Results

### 3.1. Participants’ Description

The initial sample in the pre-intervention stage comprised 221 medical doctors enrolled in one of the three experimental conditions. Of these, 85.7% (*n* =190) were women, 13.5% were men (*n* = 30), and 0.9% (*n* = 2) did not report their gender. This predominance of women is consistent with the current demographics of the Spanish healthcare system, in which women represent a growing majority among physicians. The mean age was 45.8 years (SD = 11.8). Most participants were general practitioners, 34.4% (n = 75), and their average professional experience was 18.9 years (SD = 10.8). For details on participants’ medical specialization, see Appendix A.

### 3.2. Descriptive Statistics of the Spanish Version of the SWEMWBS

To examine the psychometric properties of the Spanish version of the SWEMWBS (aim 1), we first calculated descriptive statistics for both the individual items and the total scale score. The mean total score was 25.67 (SD = 3.54), with items’ mean scores ranging from 3.15 (item 4, “I’ve been dealing with problems well”) to 4.01 (item 7, “I’ve been able to make up my own mind about things”) (see Table 1). Considering that items are rated from 1 to 5, and the global score ranges from 7 to 35, participants’ levels of well-being can be described as medium to high. No floor or ceiling effects were detected.

The first aim of this manuscript was therefore to examine the psychometric properties of the Spanish version of the Short Warwick–Edinburgh Mental Well-Being Scale in a sample of Spanish physicians. Specifically, among physicians, the WEMWBS has proven to be a useful tool for measuring well-being among professionals in China [58], Pakistan [59], or Great Britain [60]. However, there is currently no evidence regarding the performance of the shortened WEMWBS among physicians, nor in its Spanish version. The second aim is to analyze how physicians’ levels of compassion for others, professional quality of life, depression, anxiety, and stress are related to well-being. Finally, we sought to gain a better understanding of how well-being among healthcare workers may be influenced by these predictors by testing a process-based model of physicians’ well-being, grounded in both well-being theories and previous empirical evidence.

### 3.3. Internal Structure of the Spanish Version of the SWEMWBS

To further address aim 1 and provide evidence of the internal structure of the scale, a confirmatory factor analysis of a single latent factor was hypothesized. The model showed an adequate fit (see Table 2). However, the RMSEA has been shown to perform poorly in structural models with few degrees of freedom, such as the one analyzed in this study [78]. This applies to simple path models and simple CFAs, which very often have relatively few degrees of freedom. Specifically, Kenny et al. (2015) found in simulation studies that models with limited degrees of freedom (including those with a non-significant chi-square test) can still yield poor RMSEA values.

When the specific relationships within the retained model were examined, evidence of adequate factor loadings was found. Factor loadings ranged from 0.67 for item 7, “I’ve been able to make up my own mind about things”, to 0.81 for item 5, “I’ve been thinking clearly”, and all were statistically significant (*p* < 0.001) (for more details, see Table 1). No unmodeled relationships were identified in the modification indices. The pattern of the residuals was also inspected, with no evidence of model misspecification. Taking all this information into account, the model was retained as a good representation of the data.

### 3.4. Reliability Evidence of the Spanish Version of the SWEMWBS

Also addressing Aim 1, reliability estimates for the scale and its items were calculated. Cronbach’s alpha was 0.83 [95%IC = 0.79, 0.86] and McDonald’s omega was 0.89, thus providing strong evidence of reliability. Regarding the item-level’ reliability estimates, the results were also adequate. When individual items were deleted, Cronbach’s alpha decreased, and corrected item-total correlations ranged from 0.52 to 0.67. Further details are presented in Table 1.

### 3.5. Process-Based Model of Physicians’ Well-Being Using the SWEMWBS

To address aim 2, examining how physicians’ levels of compassion for others, professional quality of life, depression, anxiety, and stress are related to well-being, we tested a process-based model of physicians’ well-being grounded in both well-being theories and previous scientific evidence. As shown in Table 2, the model under study showed an adequate fit, except for the RMSEA, which performed poorly due to the low degrees of freedom, as previously discussed. However, the examination of the modification indices suggested an unmodeled relationship that could improve the model’s fit: a direct effect of compassion on well-being. This relationship was therefore included in a second, partially mediated model, which resulted in a better overall fit (see Table 2). The chi-square comparison indicated statistically significant differences between models: ∆*χ*^2^ (1) = 49.23, *p* < 0.01. This, together with an increase in the CFI (∆CFI = 0.03), supported the retention of the latter model as the best representation of the data.

**Figure 2:**
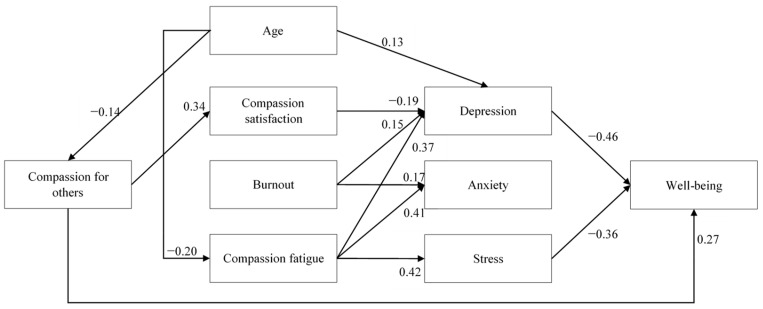
Results of the model illustrating the processes affecting physicians’ well-being. Notes: For clarity, only statistically significant relationships are displayed (*p* < 0.05). Correlations among predictors are presented in Table 3.

As shown in Figure 2, the process-based model revealed that physicians’ levels of compassion for others significantly predicted their levels of compassion satisfaction (*β* = 0.34, *p* < 0.01), but not their levels of burnout (*β* = 0.02, *p* = 0.77) or compassion fatigue (*β* = 0.04, *p* = 0.55). The dimensions of professional quality of life also predicted physicians’ emotional states. Specifically, depression was predicted by compassion satisfaction (*β* = −0.19, *p* = 0.01), burnout (*β* = 0.15, *p* = 0.04), and compassion fatigue (*β* = 0.37, *p* < 0.01); anxiety was predicted by burnout (*β* = 0.17, *p* = 0.03) and compassion fatigue (*β* = 0.41, *p* < 0.01), but not by compassion satisfaction (*β* = 0.12, *p* = 0.08); and stress was significantly predicted by compassion fatigue (*β* = 0.42, *p* < 0.01), but not by compassion satisfaction (*β* = −0.02, *p* = 0.79) or by burnout (*β* = 0.13, *p* = 0.11). Finally, well-being was predicted by depression (*β* = −0.46, *p* < 0.01) and stress (*β* = −0.36, *p* < 0.01), not by anxiety (*β* = 0.20, *p* = 0.07). Additionally, a direct impact of compassion on well-being was found (*β* = 0.27, *p* < 0.01). No statistically significant indirect effects were found.

Regarding the effects of gender and age, only three significant effects of age were observed: age positively predicted depression (*β* = 0.13, *p* = 0.01) and negatively predicted both compassion fatigue (*β* = −0.20, *p* = 0.01) and compassion (*β* = −0.14, *p* = 0.04). Correlations among the study variables are presented in Table 3. Overall, the model explained 46.7% of the variance in physicians’ well-being (*R^2^* = −0.47, *p* < 0.01).

## 4. Discussion

Mental well-being has been recognized as an essential component of health and quality of life, with major international institutions highlighting its importance for both the individual and community functioning [1,2]. Accordingly, short and reliable measurement instruments that assess their hedonic, evaluative, and eudaimonic domains are needed. One of the tools that has recently received considerable attention, due to its brevity and ease of use, is the short version of the Warwick–Edinburgh Mental Well-being Scale, which has not yet been adapted and validated in Spanish. Therefore, the first aim of this manuscript was to examine the psychometric properties of the Spanish version of the SWEMWBS in a sample of Spanish physicians.

For this purpose, responses of 221 medical doctors who were enrolled in a randomized controlled trial were analyzed. As data from the pre-intervention phase were used, no distinction between groups was made, and the sample was examined. Most participants were women and middle-aged adults. These data are consistent with those reported by the General Council of Medical Associations of Spain (*Consejo General de Colegios Oficiales de Médicos*, CGCOM) [79], which indicated that 61% of the physicians working in public health centers in Spain in 2021 were women. Thus, the higher proportion of women in this study may be attributed either to an increasing female representation in caring professions or to a greater interest in programs focused on the development of compassionate skills, given that the participants were recruited as part of a randomized clinical trial in this field. Participants’ mean age was also similar to that reported by Spanish physicians’ associations, which was approximately 49 years according to the State Confederation of Medical Unions (*Confederación Estatal de Sindicatos Médicos*, CESM) [80].

Regarding the descriptive statistics for the Spanish version of the SWEMWBS, participants’ levels of well-being were described as medium to high, consistent with previous research assessing physicians’ well-being using other instruments. For example, in the study conducted by West et al. [81], physicians’ quality of life scores averaged 6.7 points on a scale ranging from 0 to 10, prior to an intervention aimed at improving them. Similarly, other studies have also reported medium to high levels of emotional well-being, even in the context of the COVID-19 pandemic (see, for example, Capone et al. [82]). Recent interventions have also found baseline or pre-intervention flourishing levels of physicians to be around 7 points on a 0−12 scale [83]. These results suggest, from our perspective, that physicians’ levels of well-being may not be as alarming as the literature has often indicated [19,84,85]. However, since these are medium or medium-high levels, there remains considerable room for improvement through interventions targeting the variables that may predict them, as will be discussed below. This suggests that although physicians report moderate to high levels of well-being, these levels may not necessarily reflect the absence of emotional burden, but rather the coexistence of adaptive coping strategies with persistent occupational stressors, as previously discussed in the literature [15,19]. These findings are consistent with recent research reporting moderate levels of well-being among healthcare professionals in Spain, despite high workload and stress [12,86]. This pattern may indicate an adaptation to occupational demands rather than an absence of distress.

As for the results regarding the internal structure of the Spanish version of the SWEMWBS, the confirmatory factor analysis hypothesizing a unidimensional structure showed an adequate fit. The fact that the same structure was found as in studies conducted in other populations, such as Norwegian adolescents [87], Norwegian and Swedish adults [88], Scottish and Irish adolescents [89], Welsh students in years 7 to 11 [90], and the Danish adult population [52], extends the utility of the SWEMWBS for international comparison, including the Spanish version among the validated versions of the scale

With respect to reliability, coefficients were above 0.80, indicating high internal consistency. Indeed, previous studies have reported Cronbach’s alpha values ranging from 0.55 to 0.95 [55], with our findings falling around the midpoint of this range. Moreover, the reliability obtained in this study slightly exceeded the mean reliability reported in the recent reliability generalization meta-analysis conducted by Mack et al. [55], which indicated that the mean reliability estimate for the SWEMWBS was 0.81 across published studies. As for the item-level reliability estimates, all items contributed adequately to the overall reliability of the scale.

To achieve the second aim of our study, which was to examine how physicians’ levels of compassion for others, professional quality of life, depression, anxiety, and stress are related to well-being, we tested a process-based model of physicians’ well-being, grounded in both well-being theories and previous empirical evidence. In this model, compassion predicted the three dimensions of professional quality of life assessed by ProQOL, namely, compassion satisfaction, burnout, and compassion fatigue. These, in turn, predicted emotional states of depression, anxiety, and stress, as measured with the DASS-21, and these variables, in turn, predicted well-being. Gender and age were included as control variables. The mediation model that was hypothesized showed an adequate fit, but the examination of the modification indices suggested an unmodeled relationship: a direct effect of compassion on well-being. The inclusion of this relationship improved the model fit and, therefore, this second model was retained. 

Results of this process-based model revealed an initial relationship between compassion for others and compassion satisfaction. Although literature has previously pointed to positive associations between compassion and compassion fatigue (see, for example, Campos i Arnal et al. [9], in this study, compassion for others statistically and positively predicted only compassion satisfaction but not burnout or compassion fatigue. Other studies, such as that conducted by Galiana et al. [10], have also identified this relation between compassion and professional quality of life; however, in that case, the authors measured self-compassion rather than compassion for others, and professional quality of life was modeled as a single construct, with no specific effects reported between compassion and its dimensions. Similarly, studies conducted in Spain have reported that self-compassion and professional quality of life are significantly associated with well-being among healthcare professionals [12,86]. Other studies focused on the relation between compassion for others and professional quality of life have found a positive association between engagement and emotional exhaustion [34], but the relationships between compassion for others and compassion satisfaction or compassion fatigue were not examined. 

If we focus on the definition of compassion satisfaction, this dimension of professional quality of life refers to the sense of gratification derived from being exposed to and helping others through traumatic events [91], that is, the joy that comes from helping others [92]. Defined as such, it can be understood that a greater desire to alleviate the suffering of others, that is, higher levels of compassion, is associated with greater satisfaction when this relief occurs. In the same line, the lack of a relationship between compassion for others and compassion fatigue can also be explained by considering the definition of the latter construct. Compassion fatigue refers to the chronic tension and emotional exhaustion produced by continued exposure to individuals who have experienced trauma [93]; therefore, it would seem reasonable to expect that greater compassion for others might lead to higher levels of worry and fatigue. However, this may also be influenced by the magnitude and frequency with which professionals are exposed to traumatic events experienced by their patients. Thus, it would be advisable for future studies to take into account these personal experiences as a potential predictor of compassion fatigue, without assuming that trauma is equally present among all physicians. Finally, the relationship between compassion for others and burnout is less clear, although it has been previously observed in the literature [34]. Burnout is a syndrome that can be experienced by all human services professionals working in stressful situations [94], and in fact, it has been argued that, by itself, it is insufficient to explain professionals’ emotional problems arising from working with individuals who are suffering or in pain [92,93]. Thus, it appears to be the component of professional quality of life that is least related to compassion, and, by contrast, tends to develop in the presence of more general stressors or specific job-related factors, such as high workload, lack of control, insufficient recognition and reward, or lack of support and trust in the workplace, among others [95]. 

The second stage of the model tested included direct effects of the three dimensions of professional quality of life on physicians’ levels of depression, anxiety, and stress. These effects were found for depression, which was negatively predicted by compassion satisfaction and positively predicted by burnout and compassion fatigue, with the latter showing the strongest effect. That is, all three dimensions of professional quality of life contributed to the development of depressive symptoms, as previously reported in the literature. Indeed, our results coincide with those of Adhikari and Senft [24], who found that professional quality of life predicted physicians’ depressive symptoms, with compassion fatigue exerting the strongest influence. It was not the same for anxiety, which was predicted by burnout and compassion fatigue, again with compassion fatigue showing a greater impact, but not by compassion satisfaction. However, findings regarding this relationship remain inconsistent in the literature insights e, with some authors pointing a negative relation between compassion satisfaction and anxiety (i.e., Kim and Na [96], others finding no significant relationship (i.e., Hegney et al. [97]), and still others observing a positive association of compassion satisfaction with anxiety (i.e., Adhikari and Senft [24]). Finally, stress was solely predicted by compassion fatigue, with higher levels of compassion fatigue predicting higher levels of stress. Whereas the found relation has previous support [97], what is most surprising in this case is the absence of a relationship between burnout and stress. Indeed, burnout and stress have been linked since the concept of burnout was first introduced [94], and there is substantial evidence supporting this connection (see, for example, Guthier et al. [98], including evidence specific to physicians [99]. We believe that the reason why this effect was not statistically significant is due to its relatively small size: that is, this positive relationship between burnout and stress was indeed present in the data, but it was not large enough to be generalized to the population.

Finally, well-being was negatively predicted by depression and stress, but no relationship with anxiety was found. A direct and positive effect of compassion for others on well-being was also included based on the modification indices, and it proved to be statistically significant. Many authors have highlighted that psychological distress can have a significant effect on the mental well-being of healthcare professionals [15]. However, specific results relating to these two variables, distress and well-being, remain scarce, mostly due to a general misconception in the literature regarding the definition of well-being. In fact, many authors tend to equate well-being with the mere absence of psychological distress and to interpret DASS-21 scores as indicators of well-being. According to our findings, depression and stress are the components of emotional symptomatology that most strongly contribute to lower levels of physicians’ well-being. Moreover, the literature reviewed underscores the need for clearer conceptual distinction and more accurate use of the non-interchangeable concepts of emotional symptomatology and well-being. 

Finally, to conclude the discussion of the results observed in the process-based model of well-being, we would like to highlight the direct and unexpected impact that compassion for others had on the prediction and explanation of physicians’ well-being. According to the data, higher levels of compassion for others were associated with higher levels of well-being. These results align with recent evidence highlighting the role that compassion for others can have in individuals’ well-being [35], beyond the effects of sociodemographic variables, as in the work of García-Campayo et al. [35], and even after controlling for the effects of depression, anxiety, and stress, as shown by our results, which, to our knowledge, constitute the first in this regard. Previous studies have mainly focused on self-compassion rather than compassion for others [100,101]. Our findings, therefore, extend existing evidence by highlighting the potential protective role of compassion directed toward others, which has been less explored, particularly in physicians. This underscores the potential relevance of compassion-focused interventions in physicians’ training and workplace settings. In addition, the availability of a validated Spanish version of the SWEMWBS provides a valuable tool for future research, allowing researchers to monitor mental well-being across different populations and to design evidence-based interventions and policies to enhance mental health and quality of life. Taken together, these findings contribute to a growing body of evidence emphasizing the multidimensional nature of well-being.

Taken together, these findings contribute to a growing body of evidence emphasizing the multidimensional nature of well-being and its dependence on both emotional and interpersonal factors within healthcare settings

### Limitations of the Study

Several limitations should be acknowledged in the present study. The sample was limited to physicians willing to participate in a randomized clinical trial focused on compassion improvement. Therefore, these findings are difficult to generalize to other healthcare professionals, such as physicians with less interest in compassion, or professionals from other disciplines, including nurses. Indeed, the fact that those enrolled in the study volunteered to participate in a compassion-based training program could have specifically influenced the relation found between compassion for others and well-being. That is, the more relevant compassion is to an individual, the greater its potential influence on their well-being. Thus, further studies in general samples of physicians and healthcare professionals are warranted. Another limitation is the cross-sectional nature of the study, which calls for a cautious interpretation of the results, as alternative models cannot be ruled out [102]. Therefore, future research is needed to examine the interactions studied in this process-based model using a longitudinal design that makes it possible to assess the temporal relationships among these variables. Although the number of experts involved in the adaptation process of the scale was limited, they represented diverse expertise in well-being. Together with the use of a convenience sample, these aspects should be considered as potential limitations. 

Another shortcoming could be the unbalanced sample, with a greater representation of women. However, it is important to note that predominantly female samples are commonly reported in studies investigating workplace initiatives among healthcare professionals, including compassion and meditation-based intervention trials [103]. Furthermore, considering that in June 2021, 61% of physicians working in public healthcare centers in Spain were women [79], the observed percentages may reflect a greater female representation in caregiving professions and a higher interest in programs aimed at developing compassionate skills.

Finally, all the structural equation models tested showed poor values of the RMSEA. However, as stated previously, this index has shown limitations in structural models with few degrees of freedom, such as the CFA and the mediation models tested. Thus, this could be considered a limitation of the index rather than of the study itself. In Kenny et al.’s words: “Using the RMSEA to assess the model fit in models with small *df* is problematic and potentially misleading unless the sample size is very large. We urge researchers, reviewers, and editors not to dismiss models with large RMSEA values with small *df* without examining other information" [78]. Consequently, and given that both the CFI and SRMR indicated adequate fit values, we have not dismissed the models tested, despite the presence of large RMSEA values.

## 5. Conclusions

Based on the results obtained, the Spanish version of the SWEMWBS showed adequate psychometric properties and appears to be a promising tool for assessing physicians’ well-being. Nevertheless, these findings should be interpreted with caution, as they are derived from a cross-sectional study with a convenience sample and a limited number of experts involved in the adaptation process.

Additionally, this study provides evidence of a direct relationship between compassion for others and physicians’ well-being. Indeed, this is the first research to provide evidence in this regard, while controlling for sociodemographic characteristics, and the effects of professional quality of life and emotional distress (i.e., depression, anxiety, and stress). 

The results of this study carry several implications for health professionals and researchers interested in promoting health and well-being among physicians, by helping to improve interventions targeted at this goal. Prevention and intervention strategies could focus on physicians with lower compassion for others, as this may represent an entry point for the development of professional quality of life issues that can lead to emotional distress and directly compromise physicians’ well-being. It seems clear that interventions based on compassion, such as the Compassion Cultivation Training [104], the Cognitively Based Compassion Training [105], or the Compassion Focused Therapy [106], should play a key role in physicians’ education and workplace. Enhancing compassion in physicians could improve their well-being and, in turn, the quality of care they provide to their patients.

## Figures and Tables

**Table 1:** Descriptive and reliability statistics for the Spanish version of the Short Warwick–Edinburgh Mental Well-Being Scale.

Variable	Expert 1	Expert 2	Expert 3	Expert 4	CVR	M	SD	Sk	K	Min	Max	λ	$\alpha_{iid}$	cHI
Item 1	1	1	1	1	1.00	3.52	0.82	−0.46	0.07	1.00	5.00	0.73	0.80	0.57
Item 2	1	1	1	0	0.50	3.78	0.67	−0.78	1.62	1.00	5.00	0.69	0.80	0.57
Item 3	1	1	1	1	1.00	3.15	0.85	−0.30	−0.44	1.00	5.00	0.70	0.80	0.59
Item 4	1	1	1	1	1.00	3.72	0.57	−0.85	2.07	1.00	5.00	0.79	0.81	0.56
Item 5	1	1	1	1	1.00	3.66	0.69	−0.66	0.90	1.00	5.00	0.81	0.79	0.67
Item 6	1	1	1	0	0.50	3.84	0.68	−0.53	0.68	2.00	5.00	0.72	0.80	0.56
Item 7	1	1	1	1	1.00	4.01	0.64	−0.23	0.16	2.00	5.00	0.67	0.81	0.52
Total score	--	--	--	--	--	25.67	3.54	−0.48	0.17	15.00	35.00	--	--	--

Notes: M = mean; SD = standard deviation; Sk = skewness; K = kurtosis; Min = minimum score; Max = maximum score; λ = factor loading; $\alpha_{iid}$ = Cronbach’s alpha if item deleted; cHI = corrected homogeneity index.

**Table 2:** Results of the structural equation models tested.

Model	$\chi^{2}$	*df*	p	CFI	SRMR	RMSEA	90%IC
Confirmatory factor analysis	152.37	14	<0.01	0.92	0.07	0.22	0.19, 0.25
Total mediation model	68.99	7	<0.01	0.91	0.07	0.20	0.16, 0.45
Partial mediation model	49.23	6	<0.01	0.94	0.05	0.18	0.14, 0.23

Notes: *df* = degrees of freedom; CFI = Comparative Fit Index; SRMR = Standardized Root Mean Square Residual; RMSEA = Root Mean Square Error of Approximation; IC = interval of confidence.

**Table 3:** Correlations with the model for the processes affecting physicians’ well-being.

Variables	Compassion Satisfaction	Burnout	Compassion Fatigue
Compassion satisfaction	--		
Burnout	−0.48	--	
Compassion fatigue	−0.47	0.69	--
**Variables**	**Depression**	**Anxiety**	**Stress**
Depression	--		
Anxiety	0.52	--	
Stress	0.42	0.62	--

Notes: All correlations resulted in statistically significant (*p* < 0.01).

## Data Availability

The datasets presented in this article are not readily available because the data are part of an ongoing study. The data are available from the authors upon reasonable request.

## Abbreviations

The following abbreviations are used in this manuscript:

SWEMWBS	Short Warwick–Edinburgh Mental Well-Being Scale
WHO	World Health Organization
OECD	Organization for Economic Co-operation and Development
SWLS	Satisfaction With Life Scale
PANAS	Positive Affect and Negative Affect Scale
SPANE	Scale of Positive and Negative Experiences
RPWB	Ryff’s scales of Psychological Well-Being
WHO-5	WHO Well-being Index
WEMWBS	Warwick Edinburgh Mental Well-Being Scale
CVR	Content Validity Ratio
CVI	Content Validity Index
CCTW	Compassion Cultivation Training Workshop
MBI	Mindfulness-Based Intervention
NI	No-Intervention Group
MANOVA	Multivariate Analysis of Variance
DASS-21	Depression, Anxiety, and Stress Scale
ProQOL	Professional Quality of Life
SOCS-O	Sussex–Oxford Compassion for Others Scale
CFI	Comparative Fit Index
SRMR	Standardized Root Mean Square Residual
RMSEA	Root Mean Square Error of Approximation
WLSMV	Weighted Least Squares Mean and Variance-Corrected
MLR	Maximum Likelihood with Robust Standard Errors
CGCOM	Consejo General de Colegios Oficiales de Médicos
CESM	Confederación Estatal de Sindicatos Médicos

## Appendix A. Participants’ Medical Specialization

**Table A1:** Participants’ medical specialization.

Medical Specialization	n	%
Family and Community Medicine	75	33.6
Pathological Anatomy	1	0.4
Anesthesiology and Resuscitation	10	4.5
Digestive System	2	0.9
Cardiology	3	1.3
General and Digestive Surgery	1	0.4
Orthopedic Surgery and Traumatology	1	0.4
Medical-Surgical Dermatology and Venereology	7	3.1
Endocrinology and Nutrition	2	0.9
Geriatrics	6	2.7
Hematology and Hemotherapy	4	1.8
Medicine, Physical Education, and Sport	1	0.4
Intensive Care Medicine	7	3.1
Internal Medicine	9	4.0
Preventive Medicine and Public Health	1	0.4
Occupational Medicine	3	1.3
Microbiology Parasitology	1	0.4
Nephrology	4	1.8
Pneumology	1	0.4
Clinical Neurophysiology	1	0.4
Neurology	9	4.0
Obstetrics and Gynecology	15	6.7
Medical Oncology	7	3.1
Radiation Oncology	1	0.4
Otorhinolaryngology	6	2.7
Pediatrics	14	6.3
Psychiatry	13	5.8
Radiodiagnostics	1	0.4
Rehabilitation	2	0.9
Rheumatology	2	0.9
No specialty	5	2.2
Other specialty	4	1.8
Missing	4	1.8
